# Microbial ecology‐based engineering of Microbial Electrochemical Technologies

**DOI:** 10.1111/1751-7915.12802

**Published:** 2017-08-14

**Authors:** Christin Koch, Benjamin Korth, Falk Harnisch

**Affiliations:** ^1^ Department of Environmental Microbiology Helmholtz Centre for Environmental Research GmbH ‐ UFZ Permoserstraße 15 04318 Leipzig Germany

## Abstract

Microbial ecology is devoted to the understanding of dynamics, activity and interaction of microorganisms in natural and technical ecosystems. Bioelectrochemical systems represent important technical ecosystems, where microbial ecology is of highest importance for their function. However, whereas aspects of, for example, materials and reactor engineering are commonly perceived as highly relevant, the study and engineering of microbial ecology are significantly underrepresented in bioelectrochemical systems. This shortfall may be assigned to a deficit on knowledge and power of these methods as well as the prerequisites for their thorough application. This article discusses not only the importance of microbial ecology for microbial electrochemical technologies but also shows which information can be derived for a knowledge‐driven engineering. Instead of providing a comprehensive list of techniques from which it is hard to judge the applicability and value of information for a respective one, this review illustrates the suitability of selected techniques on a case study. Thereby, best practice for different research questions is provided and a set of key questions for experimental design, data acquisition and analysis is suggested.

## Introduction

Since the turn of the millennium, microbial electrochemical technologies (MET) advanced from the lab‐bench to technical scale (Schröder *et al*., 2015). First prototypes, especially of microbial fuel cells (MFCs) and microbial electrolysis cells (MEC), were installed, and commercialization seems in reach (Gil‐Carrera *et al*., 2013; Brown *et al*., 2014; Heidrich *et al*., 2014). At the same time, the detailed understanding and knowledge‐driven engineering of the MET components and especially their complex interplay are differently elaborated. Among others, major foci have been on the engineering of electrode materials (Kipf *et al*., 2014; Baudler *et al*., 2015) and architecture (Logan *et al*., 2015), membranes and separators, reactor design (He *et al*., 2016) and benchmarking (Harnisch and Rabaey, 2012; Patil *et al*., 2015) as well as striving for optimization and integration of processes. However, composition, activity and dynamics of the microorganisms that interact with the electrodes have been rather an aspect of posterior and only selective characterization than active management, for example, Kiely *et al*. (2011), Ishii *et al*. (2014). In other words, the ecology and physiology of the involved microbial communities that form the beating heart of every MET is largely untapped.

The wiring of the microbial metabolism and the current flow at an electrode forms the fundament of all MET. For model organisms like *Geobacter* and *Shewanella*, the details of the interactions between microorganisms and electrodes on the cellular as well as subcellular level are getting increasingly understood (Lovley, 2012; Richter *et al*., 2012; Kumar *et al*., 2017). However, aside from these model organisms, this is not the case (Koch and Harnisch, 2016). Especially for electroactive microbiomes, comprising biofilms as well as planktonic cells, that are enriched from complex inocula (e.g., wastewater) only very little is known. At the same time, non‐sterile operation is considered for the majority of future application scenarios, and hence electroactive microbiomes rather than pure cultures are suggested to drive these processes (Marshall *et al*., 2013; Angenent *et al*., 2016). Therefore, knowledge on the individual microbe‐electrode interaction, the microbial interactions within the biofilm, and the interactions between the biofilm and the surrounding planktonic cells are needed to proactively steer microbiome‐based MET (e.g., for valorizing waste and wastewater). Alike to other processes like anaerobic digestion (AD), it seems obvious that the microbial communities in MET form a complex metabolic network allowing the utilization of complex substrates mixtures (Vanwonterghem *et al*., 2014). These microbial communities cannot be described by the simple term biomass or using other classical parameters relevant in pure culture biotechnology (e.g., cell number, dry mass). In contrast, one can consider this complex network of microorganisms and microbial interactions being similar to those found in ecological systems on the macroscopic level. For instance, those complex food webs and interactions are found between the different groups of animals covering insects, small primary consumers of plant material, secondary consumers, up to carnivorous species forming the end of the food chain (Kormondy, 1996). Although being already very complex in terms of interaction of individuals, we are at least able to directly observe these individuals and interactions in most cases. It is rather a question of time and patience for analysing the food preferences as well as collecting or hunting strategies of a species. This is very different for analysing microorganisms and their interactions on the microscopic scale. The size of an average microbial cell is in the range of 1 μm and, let's be honest, most bacteria look pretty much alike under the microscope. Further, functions or food preferences of different microorganisms can hardly be derived from microscopic images. The diversity of animal and plant species, their complex food webs and interactions, that is termed ecology, have been investigated and described in detail for ages (Kormondy, 1996). In contrast, the field of microbial ecology is still relatively young (Rittmann, 2006), especially when compared with the general research performed in the field of microbiology which is usually focused on questions of clinical relevance. In addition to their significance for natural elemental cycles and human health, microbiomes can also form the basis for many biotechnologies (Marshall *et al*., 2013; Verstraete, 2015). Noteworthy, microbial ecology not only characterizes the diversity of microorganisms but also describes the unifying principles of their interaction, their activity and their dependency on the physical and chemical environment (Konopka, 2009). Thus, microbial ecology is the key for understanding and subsequent engineering and managing microbiomes as well as their functions.

For a comprehensive understanding and engineering of MET, we need to unravel the ecology of the involved microbiomes and the underlying patterns that determine their function in terms of application relevant parameters (Koch *et al*., 2014a). The term microbial ecology has been linked to microbial electrochemical technologies in the past (e.g., Rittmann (2006), Rabaey *et al*. (2007)) but compared to the number of articles published annually in the field, only a minority considers electroactive microbiomes and even fewer the complex underlying microbial interactions. On the one hand, this might be addressed to a limited awareness of the relevance of ecological principles for industrial application but, on the other hand, might be also due to methodological limitations. Microbial ecology provides an arsenal of techniques targeting different phylogenetic and functional levels. However, it is not always clear which methodical approach is suited best to answer a specific research question properly. To reduce this gap, this review provides insights into the relevance of microbial ecology for the characterization as well as future engineering and management of microbial electrochemical technologies. Instead of providing a comprehensive list of techniques from which it is hard to judge the applicability and information value for a respective one, the suitability of the most common techniques will be illustrated on a selected case study (Pant *et al*., 2013). Based on this case study, recommended techniques and the possible derived insights are discussed. Therefrom, general recommendations are deduced for applying the principles of microbial ecology on future engineering of MET.

## The MET as black box: What can be directly measured and what not?

**Figure 1:**
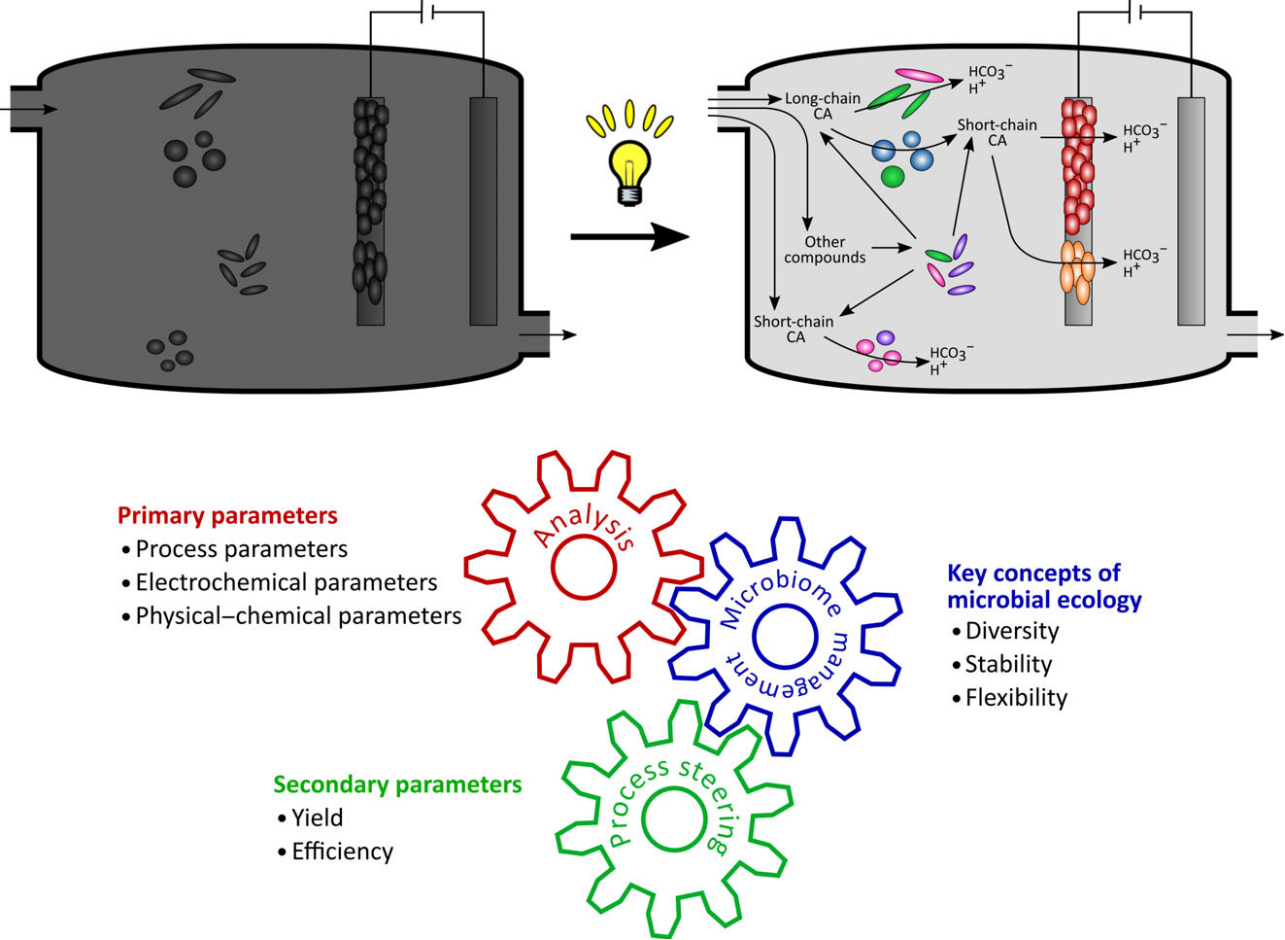
Schematic representation of the potential of microbial ecology for the analysis and engineering of microbial electrochemical technologies. Although a strong interconnection between primary and secondary parameters exists, microbial bioelectrochemical systems (BES) are often considered as ‘black box’. That means that the involved microorganisms, their phylogenetic background, activity status and their individual functional contributions are unknown. Thus, process engineering can only be performed on trial and error basis which is time and resource demanding. In contrast, targeted microbiome analysis can reveal structure–function relationships allowing proactive microbiome management. With the adequate choice of techniques, it can be disclosed, for instance, which are the main degraders of a complex substrate and carboxylic acids (CA) as intermediates (depending on substrate choice and degradation pathways). Further, it can be revealed if the conversion is performed (mainly) in the bulk liquid or at the anode and which biological requirements and limitations regarding primary parameters exist. By harnessing key concepts of microbial ecology, the process of interest can be steered. For simplicity, the figure represents only a schematic example of a BES process and not all possible reactions and interconnections (e.g., specific substrates, presence of alternative electron acceptors, syntrophic interactions) are included.

Considering a bioelectrochemical system (BES) designed for a certain MET application (e.g., wastewater treatment and current production in a MFC), a certain set of primary parameters like process parameters, electrochemical and physical–chemical parameters (Fig. 1) can be directly measured but is not always comprehensively reported (Patil *et al*., 2015). Based on these primary parameters, benchmarking parameters of the respective processes can be derived (secondary parameters, Fig. 1). However, some highly important features or properties of MET can neither be described nor engineered without taking the impact of the microorganisms into consideration (key concepts of microbial ecology, Fig. 1, Box 1).

Box 1 **Selected key concepts in microbial ecology with relevance for engineering of microbial electrochemical technologies:**

**Diversity**

A diverse microbial community is more likely to provide a higher number of physiological capacities that, for instance, enable the community to successfully utilize substrate mixtures. A high diversity is often seen as guarantor for a stable process function under varying operational conditions. In contrast, maximum performance, in terms of efficiency and velocity, is often the result of a reduced diversity.

**Stability**

Stability is often considered as maintaining the process function from an engineer's perspective. This can be realized by a stable microbial community composition but also by a flexible one which successfully adapts to changes in primary parameters and ensures a stable functionality by variations of individual activities.

**Flexibility**

Flexibility is the capacity of the microbial community to adapt to changes in the primary parameters. Depending on the process, a high flexibility can be required to cope with, for example, fluctuating substrate inflows regarding amount and composition (e.g., domestic wastewater). Other processes are characterized by highly stable primary parameters and can therefore replace the requirement for high flexibility with the focus on maximum performance. A high diversity of the microbiome usually provides higher flexibility than a specialized microbiome with low diversity.

## Application of microbial ecology to MET

**Figure 2:**
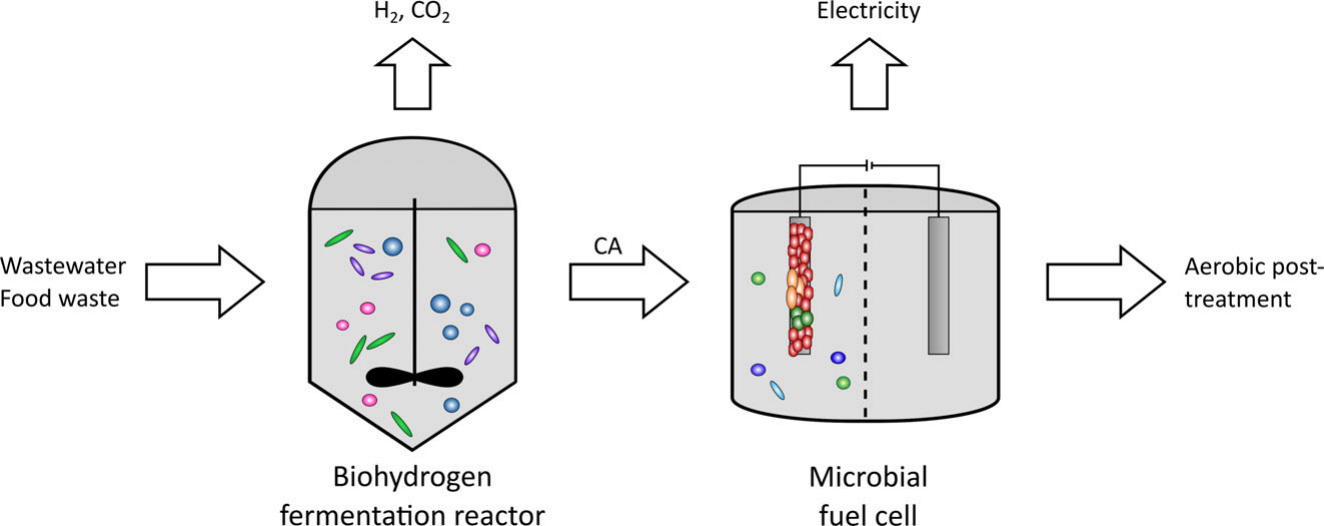
Process scheme of the case study: A biohydrogen fermentation reactor and a microbial fuel cell (MFC) are coupled for the combined treatment of wastewater and concentrated organic food waste in a two‐step process (Pant *et al*., 2013). The fermentation products (carboxylic acids, CA) of the bioreactor are transferred to the MFC, and there are further degraded, and electricity is produced. Further process details are given in (Pant *et al*., 2013). The figure visualizes the involved compartments. A potential respective analysing strategy based on microbial ecology methods is described in the text.

In the following, the principles of deriving information from microbial ecological analysis on MET are shown. This information can form the fundament of a microbiome‐based management of BES to reach performance targets (see Fig. 1). Microbial ecology provides an arsenal of techniques targeting different phylogenetic and functional levels that result in a different depth and type of information (see Table 1). For a non‐expert in microbial analysis and ecological interpretation, it can be hard to judge which technique provides the most valuable answer for MET improvement in the light of a specific research question. The complex outcome of a microbiome analysis has to be combined with engineering parameters and an appropriate interpretation. To guide the reader and potential operator, we chose a case study for illustrating a microbial ecology‐based approach. ‘Integrated conversion of food waste diluted with sewage into volatile fatty acids through fermentation and electricity through a fuel cell’ by Pant *et al*. (2013) demonstrates treatment and valorization of organic wastes including biohydrogen production by fermentation and electric energy generation by a MFC (Fig. 2). The paper published as it is does not include any microbial analysis. Based on this case study, we discuss suitable microbiome analyses based on microbial ecology, their potential outcomes and interpretations as well as limitations to guide future operators for their choice of methods and provide best practice recommendations.

**Table 1:** Selected techniques of microbial ecology that are suitable for characterization of MET

Technique	Principle & marker	Information	Selected recommended references and examples
16S rRNA gene sequencing	The DNA of the microbial sample is extracted, and the 16S rRNA gene is partially amplified and sequenced. Depending on the applied approach this can cover a suitable small region, for example, in the range of 100‐200 bp dependent on choice of primers, (Liu *et al*., 2007; Soergel *et al*., 2012; Klindworth *et al*., 2013).	The obtained results can be interpreted in a phylogeny dependent as well as independent way. For the first, the obtained sequences are compared to databases (e.g., using the Ribosomal Database Project (Cole *et al.,* 2014)). The sequences are assigned on different phylogenetic levels going down to genera, dependent on the respective sequences, their length and the available information in the databases. For phylogeny independent analysis, individual sequences are grouped into OTUs (e.g., 97% sequence identity although this is not always representative of a single species). The diversity of the microbial community sample is then characterized based on the number of different phylogenetic groups or OTUs (richness) and their relative abundances (evenness). The technique is suitable to get a general impression of the phylogenetic composition of a microbial community and allows monitoring reactor microbiomes over time and in response to changes in process parameters.	Gilbert *et al.,* (2012); Ishii *et al.,* (2014); Yarza *et al.,* (2014)
16S rRNA gene fingerprinting (e.g. T‐RFLP)	The DNA of the microbial sample is extracted, and the 16S rRNA gene is partially amplified. Depending on the applied approach this can cover nearly the complete gene (Schütte *et al*., 2008; Lefebvre *et al*., 2010). Depending on the chosen technique, the fingerprint consists of fragments which are representative of sequence characteristics, for example, position of cutting sites for restriction endonucleases.	The obtained results are interpreted in a phylogeny independent way. The fragments of the fingerprint represent a certain group of organisms that have similar sequence characteristics, for example, the same position of a cutting site for a restriction endonuclease. Each fragment (e.g., peak in a T‐RFLP profile) represents one OTU. The diversity of the microbial community sample is then characterized based on the number of different OTUs (richness) and their relative abundances (evenness). The technique is suitable to get a general impression of the diversity of a microbial community and allows monitoring reactor microbiomes over time and in response to changes in process parameters although phylogenetic information is not provided.	Marzorati *et al.,* (2008); Schütte *et al.,* (2008); Koch *et al.,* (2014b)
Cytometric fingerprinting	Cytometric fingerprinting is a single cell‐based approach that utilizes optical characteristics (cell size, DNA content after staining) of individual microbial cells to characterize a microbial community sample.	The optical characteristics are independent of the phylogenetic background of the cells. Complex microbial communities are characterized in a simple and rapid way. The changes in the cytometric fingerprint are, similar to 16S rRNA fingerprinting, representative of changes in the community composition and allow monitoring reactor microbiomes over time and in response to changes in process parameters.	Koch *et al.,* (2013); Günther *et al.,* (2016)
Metagenomics, Metatranscriptomics, Metaproteomics	The entire DNA, RNA or expressed protein content of a microbial community is analysed.	The results reflect the genes and their expression products that reveal the presence of certain metabolic capacities. Including also abundance information, potential metabolic pathways in the microbial community can be identified and allocated to individual species.	Ishii *et al.,* (2013); Wöhlbrand *et al.,* (2013); Vanwonterghem *et al.,* (2016)
Fluorescence *in situ* hybridization (FISH)	FISH is a single cell‐based approach that utilizes phylogenetic information in form of a target specific, fluorescently labelled probe that hybridizes to the DNA or RNA within the cells. Therefore, a priori knowledge about the potential relevant microorganisms in a microbial community (e.g., 16S rRNA gene sequencing) is recommended.	The technique allows detection and enumeration of bacteria based on a specific phylogenetic marker and can reveal the spatial organization of the cells, for example, cell density and different layers within a biofilm.	Amann and Fuchs (2008); Mielczarek *et al.,* (2013); Shrestha *et al.,* (2013)
NanoSIP/nanoSIMS	The assimilation of substrates marked with stable isotopes (e.g., ^13^C, ^15^N, ^34^S or ^2^H) in microbial biomass is visualized on single cell level in combination with a phylogenetic marker. A priori knowledge about the potential relevant microorganisms and their potentially utilized substrates is recommended.	The results reflect metabolic activity in combination with phylogenetic as well as spatial information on single cell level.	McGlynn *et al.,* (2015); Musat *et al.,* (2016)
Electrochemical microcosm	Small scale BES can be set up for characterizing specific functions of electroactive biofilms.	Under defined conditions, the microbial activity can be investigated including utilization of specific substrates as well as detailed mechanisms of the microorganism–electrode interaction.	Pous *et al.,* (2014)

### The case study – an engineer's viewpoint

Pant *et al*. (2013) described the combined treatment of wastewater and concentrated organic food waste in a two‐step process. They coupled a bioreactor for the fermentative production of hydrogen and carboxylic acids (CA) from combined wastes with a microbial fuel cell for further degradation of acids and electricity production. The combined treatment reduced the chemical oxygen demand (COD) of the process liquid by 90%. This successful demonstration of a potential process was characterized by the authors based on primary parameters (e.g., concentration of fermentation products, power density) and the impact of changes of operational parameters (e.g., hydraulic retention time, pH) on the system performance (i.e., secondary parameters like COD removal and coulombic efficiency). All experiments were run under mesophilic conditions. No microbial characterization was performed.

The original feedstock can be considered as complex, diverse substrate as it contains domestic wastewater and a defined mixture of food waste (apple, pear, banana, lettuce, carrot, tomato, potato, bread, coffee filter paper, egg and pork meat; Pant *et al*. (2013)). Due to the diverse but defined mixture of the food waste, the substrate entering the first reactor can be considered as being relatively stable in its composition. In the first reactor, hydrolysis and acidogenesis took place leading to the formation of carboxylic acids and hydrogen (Fig. 2). The effluent of this reactor was collected and diluted with phosphate buffer to stabilize the pH and reach a chemical oxygen demand of 1.200 mg l^−1^ before being inserted into the MFC. The general composition of the substrate mixture entering the MFC anode compartment in the consecutive batches was similar in terms of types of carboxylic acids (acetic acid, propionic acid, butyric acid, isobutyric acid, isovaleric acid, valeric acid, isocapronic acid, capronic acid) but differed in the individual concentrations of each compound (details table 4 in Pant *et al*. (2013)). Highest total concentrations of carboxylic acids were reached with 417 mg l^−1^. The highest individual contributions per batch were acetic acid (276 mg l^−1^) and butyric acid (147 mg l^−1^). After MFC treatment, the total concentration of carboxylic acids was below 11 mg l^−1^.

This study nicely demonstrates that the general set‐up of a treatment strategy for organic wastes can be successfully performed without any knowledge on the involved microorganisms. However, in the following, we discuss which microbial analyses could have been performed for this representative experimental set‐up to understand the underlying microbial interactions and to improve MET based on microbiome management. Especially, we focus on the accurate conduction of certain techniques to derive valuable information, their potential outcomes, the functional interpretation of data in the eye of a microbial ecologist and their limitations to foster a microbial ecology‐based engineering of MET.

### A bioreactor as an ecological niche

In the case study, hydrolysis and acidogenesis take place in the first reactor representing specific functions for waste valorization (Fig. 2). These functions can be performed by a variety of microorganisms. Pant *et al*. had chosen a highly diverse inoculum for this reactor based on green waste and kitchen waste compost. Generally speaking, the more diverse the inoculum the higher is also the probability to introduce microorganisms into the reactor that perform the desired functions under the respective conditions. From an ecological point of view, the specific process environment of a reactor is an ecological niche (see also Box 2). In this ecological niche, the microorganisms face a defined temperature, substrate composition and concentration, pH, etc. Those microorganisms that can perform best in terms of reproduction in this niche will utilize the provided substrates first, increase their cell number and finally occupy the niche. This does not automatically mean that the dominating microorganisms are also the microorganisms that show the best performance from an operator's point of view regarding a required function. However, the operator can set the boundary conditions (i.e., thermodynamic limits) and hence try to shape the process environments to foster the respective microorganisms (Hanselmann, 1991).

Box 2 **Glossary**

- *16S rRNA gene*: This gene encodes the RNA of the small ribosomal subunit which is present in all bacteria. It has a length of about 1500 base pairs (bp) and is characterized by the presence of highly conserved and highly variable regions that serve as well‐established phylogenetic marker for diversity analyses. The sequence information itself is often used in an already classified way by determining sequences with more than 97% identity as the same species. Alternatively, indirect sequence characteristics can also be utilized, for example, the length of restriction fragments in T‐RFLP (terminal restriction fragment length polymorphism) – an established DNA‐based fingerprinting method.
- *Diversity*: Diversity characterizes the structure and composition of a microbial community in terms of number of different species (richness) and their relative abundance (evenness). The term is established in classical ecology and often seen as guarantor for stable function (resilience) under varying environmental conditions (e.g., temperature change as a result of climate change).
- *Ecological niche*: An ecological niche is a specific locality in which an individual organism is exposed to a range of environmental conditions that allow the individual to persist and utilize the present resources (Schoener, 2009). These conditions determine if microorganisms can exist and how they interact with their environment (Losos, 2009).
- *Electroactive microbiome*: A diverse microbial community that is in its entity able to interact with an electrode. In the case of anodic processes, this electroactive microbiome is able to degrade organic matter and transfer electrons to the electrode as terminal electron acceptor. The functional capacities are differentially distributed between the microorganisms within the microbiome. This means that not all cells are electroactive or show this capacity when cultivated as pure culture. Further, trophic networks are very likely to be formed between the microorganisms leading to emerging functions of the microbiome that none of the involved microorganisms could realize on its own.
- *Metagenome*: The entire set of sequences and genes, respectively, representing a microbial community sample. Depending on sequencing technology, this can be gigabase pairs (10^12^ bp) of DNA per sample and sometimes even complete genomes can be extracted dependent on the diversity of the sample and individual coverage.
- *Operational taxonomic unit (OTU)*: An OTU is an arbitrary unit resulting from diversity analyses. Especially, fingerprinting techniques result in certain fragments that depend on the sequence characteristics of the underlying DNA (see also 16S rRNA gene). These fragments represent a certain group of organisms that have similar sequence characteristics, for example, the same position of a cutting site for a restriction endonuclease. But it does not automatically mean that the fragments represent the same species. Often, this is the case but sometimes also very different species can have the same restriction sites also depending on the chosen restriction enzyme. Nevertheless, OTUs give a good measure of the diversity in a sample, as the number of different OTUs is comparable when the same restriction endonuclease has been applied. Besides the number of OTUs, also their abundance and evenness can be considered being representative measures of diversity.
- *Relative abundance*: The abundance of an organism or an OTU, for example, based on 16S rRNA, in a sample can only be determined in a relative way because the individual sequence or OTU is compared to the overall number of OTUs in a sample. The DNA extraction efficiency from different species can be significantly different due to differences in their cell wall characteristics and dependent on the extraction method. Also differences in DNA amplification efficiency and target copy number per cell exist. Nevertheless, these relative abundances can reveal clear trends in community composition and dynamics, but the potential for not detecting certain community members should also be considered, especially if new environments or substrates are explored (Kuczynski *et al*., 2011).
- *Selection (pressure)*: A selection pressure is a specific combination of environmental conditions (see also ecological niche) that enhances or suppresses the growth of organisms compared to others under specific circumstances. For instance, *Geobacter* sp. is strongly selected in a BES inoculated with primary wastewater, an anode potential of 0.2 V versus Ag/AgCl sat. KCl, and running with acetate as sole carbon source.

The high diversity of the inoculum will decrease due to the specific process conditions provided in this niche. In the case study, the microorganisms adapted to higher temperatures – the kitchen waste was derived from thermophilic compost – will probably not survive or be at least substantially limited in their activity in the mesophilic reactor environment which does not meet their individual physiological requirements. But still, the microbial community will have a high diversity due to the substrate's complex and diverse composition. The mixture of CA is subsequently introduced into the MFC (Fig. 2). The degradation of CA can be performed by numerous organisms. They can either degrade the long‐chain carboxylic acids to short‐chain carboxylic acids through fermentation (acetogenesis) or utilize acetate by anaerobic oxidation if final electron acceptors are available. These final electron acceptors can be provided in form of an electrode, syntrophic partner (e.g., hydrogen scavenging microorganism and direct interspecies electron transfer) or other soluble (e.g., nitrate or sulfate) and insoluble electron acceptors (e.g., humic substances, metal oxides) (Koch and Harnisch, 2016). Overall, the microbial diversity very likely differs from the diversity of the biohydrogen fermentation reactor as completely different substrates are metabolized. In contrast to the first reactor, which is completely mixed, a spatial heterogeneity can be expected in the MFC. Some cells use the anode as terminal electron acceptor, colonize the anode and form a biofilm. Other cells might also preferentially reside in a biofilm although not being metabolically connected to the electrode leading to biomass retention (De Vrieze *et al*., 2014). Other microorganisms especially those performing fermentation are very likely present as planktonic cells. In this way, a specific and functional food web can be built in both reactors. Due to the complexity of the provided substrate as well as the high diversity of the inoculum, this food web is very likely to be flexible towards changes in its environment, for instance, in terms of substrate composition and organic load (see detailed discussion section ‘From reactor description to improved MET engineering’).

### Community composition based on phylogenetic markers

Today's routine approach for microbial characterization of comparable technical systems and microbial communities in general is the diversity analysis on 16S ribosomal RNA (rRNA) level (Table 1). *What would we expect to find for the case study if the biohydrogen fermentation reactor (planktonic cells) and the MFC (planktonic cells, biofilm) were sampled regularly over the course of the experiment?*

At start of the reactor operation, the microbial community of the biohydrogen fermentation reactor can be assumed to be identical to the community of the inoculum. But its composition adapts very rapid in response to the substrate and reactor environment provided. As already discussed earlier, an enrichment of the microorganisms that are adapted best to the provided ecological niche in the reactor will take place. This enrichment will reduce the microbial diversity compared to the inoculum in terms of number of operational taxonomic units (OTUs, see Box 2 Glossary), thus lowering the richness but still being diverse due to the complex and diverse substrate. The evenness, that is the comparative abundance of the individual OTUs, in the microbial community will still be relatively high as no single species is expected to solely drive the degradation of the complex and diverse substrate. Considering also phylogenetic information, the biohydrogen fermentation reactor community will probably be dominated by *Firmicutes* and *Bacteroidetes* being well known for their capacity for hydrolysis and acidogenesis (Kim *et al*., 2010; Guo *et al*., 2014; Yi *et al*., 2014). Methanogenic archaea will probably be detectable in the inoculum but will disappear during the process as the experiments were performed below pH 6 and with short hydraulic retention times. Under these conditions, the activity of methanogenic archaea is usually limited and their growth inhibited (Kim *et al*., 2004; Pakarinen *et al*., 2011). Accordingly, they will be outcompeted and diluted over time. During the experiment, the bacterial community composition will remain, but variations in the individual abundances will probably appear due to small variations in the substrate composition during the course of reactor operation over time.

By transferring the process liquid, the microbial community of the biohydrogen fermentation reactor is inserted into the MFC, which has been pre‐enriched with microorganisms. The microbial enrichment in the MFC was based on sodium acetate as the only source of carbon and electrons and the anode as only electron acceptor. Acetate in combination with a poised electrode at positive potential represents a strong selection pressure for the enrichment of *Geobacter* sp. on the anode (Harnisch *et al*., 2011). Depending on the time of enrichment, the applied potential, and original inoculum, a thick reddish biofilm is formed on the anode within 2 weeks while the reactor liquid is not turbid indicating that there is a minor number of planktonic cells. Using molecular analysis, phylogeny independent techniques would show a clear increase of abundance of a single OTU (e.g., a dominant peak in T‐RFLP), sometimes even an OTU which was below the detection limit in the inoculum. Using sequence‐based analyses, this OTU could be affiliated to *Geobacter* sp. and the family *Geobacteraceae*, respectively. For the MFC of the case study, the pre‐enrichment time is not given, but it is very likely that the ecological niche of the electrode was already occupied by a *Geobacter*‐dominated biofilm before the reactor community of the biohydrogen fermentation reactor entered the system. As already mentioned before, the microbial community entering the MFC was already shaped towards the ecological niche provided in the biohydrogen fermentation reactor. This niche is different to that provided in the MFC: While still being diverse, potentially functional microorganisms relevant for the MFC could have already been outcompeted during residence in the biohydrogen fermentation reactor. This is also advantageous for the exclusion of methanogenic archaea in the MFC, but slow growing syntrophic microorganisms for the degradation of organic acids might have been lost already. In the case study, the short hydraulic retention time and the subsequent collection of the entire effluent might have avoided the complete elimination of the low abundant species with low activity and long‐generation times, respectively. Further, the pre‐selection for *Geobacter* sp. on the electrode might also limit the metabolic potential of the microbial community as other microorganisms can only secondarily colonize the biofilm. When now the microbial community of the biohydrogen fermentation reactor enters the MFC, a strong change in the microbial community composition of the planktonic cells can be expected. This adaptation would be characterized by a shift in the composition of OTUs and by the presence of *Clostridia* (e.g., *Syntrophomonas*), *Bacteroides* and *Proteobacteria* (Li *et al*., 2013; Ishii *et al*., 2014) on the sequence level. Comparing the planktonic cells to the biofilm, other differences can also be expected. As discussed previously, the biofilm will be characterized by a low diversity and the dominance of a single OTU. As *Geobacter* can only grow with the anode as final electron acceptor in the environment provided by the reactor set‐up, it can be expected that *Geobacter* is absent in the planktonic community or only present in very low abundance. Over time, an increased contribution of other OTUs in the biofilm is possible as other species might colonize the existent biofilm, either to use it as surface for biomass retention or for a direct metabolic interaction with the electrode. The latter might be less likely because the anode is already covered with a primary biofilm of electroactive species. Alternatively, a metabolic interaction of planktonic cells with the electroactive species in the biofilm, that is based on the utilization of metabolites, can take place and can be considered more likely.

### Community composition based on functional markers

So far, only the phylogenetic diversity of the microbial community was considered. Combined with a regular sampling over time covering planktonic cells in the biohydrogen fermentation reactor and the MFC as well as biofilm cells in the MFC, a first impression on major phylogenetic groups and the stability of the composition can be gained (see also Table 1). However, a functional contribution of the individual groups based on their phylogenetic affiliation cannot be easily derived from this data. For example, the genus *Clostridium* (Family *Clostridiaceae*, phylum *Firmicutes*) comprises mainly gram positive, anaerobic and spore forming microorganisms that are metabolically diverse and probably present in the described biohydrogen fermentation reactor of the case study. Most of them are chemoorganotrophs (they derive energy and carbon from organic compounds), some species are able to perform CO_2_ fixation, some others utilize inorganic compounds for energy generation, and some can even fix atmospheric nitrogen (Bergey's Manual of Systematic Bacteriology, 2009). They can be saccharolytic, proteolytic, neither or both, and produce mixtures of organic acids and alcohols from the different individually preferred substrates (Bergey's Manual of Systematic Bacteriology, 2009). This high metabolic diversity within the genus *Clostridium* cannot be mirrored based on a short 16S ribosomal RNA sequence; thus, the presence of the genus can be hardly interpreted regarding any potential function. Accordingly, supplementary methods are required for functional insights into microbial communities.

While specific amplification of functional genes has been widely applied in the past, metagenomics, transcriptomics and proteomics are more common today (Table 1). They provide information on the complete DNA, RNA or protein compositions of a sample covering, for example gigabase pairs (10^12^ bp) of DNA for each sample (sometimes even complete genomes can be extracted). In this way, these methods also capture rare species as well as their genes and proteins, respectively. By comparing the derived data sets with databases of annotated genes and proteins (Kanehisa *et al*., 2016; The UniProt Consortium, 2017) putative functions of the microbial community can be deciphered. While the presence of a functional gene cannot guarantee its actual expression and functional contribution, the measurement of the transcribed messenger RNA (mRNA) or even the expressed proteins (proteomics) gives a better and more realistic picture of actual functions in a microbial community (Ram *et al*., 2005; Wöhlbrand *et al*., 2013; Embree *et al*., 2015).

In the case study, the biohydrogen fermentation reactor would probably be characterized by numerous different fermentation pathways represented by the presence of hydrolases (e.g., glycosidases and proteases) and hydrogenases for breaking down the complex substrate mixture and hydrogen production (Mohd Yasin *et al*., 2011; Cabrol *et al*., 2017). Dependent on their specificity, they could be affiliated to a distinct phylogenetic group, for example, the above already mentioned *Firmicutes* and *Bacteroidetes*. In the MFC, the planktonic community will be characterized by further fermentative pathways while in the biofilm a clear dominance of electrode respiration related genes and proteins like cytochromes located in the outer membranes will be present (Ding *et al*., 2006; Nevin *et al*., 2009). Dependent on the microbial diversity within the samples, the presence of certain groups of genes or proteins can also enable the reconstruction of potentially realized metabolic pathways or species interaction on a trophic level (Embree *et al*., 2015; Vanwonterghem *et al*., 2016). In the case study, the diversity would probably be too high for deciphering this network using the above mentioned methods as different species can perform the same functions (functional redundancy) and species specific interactions not derived. Therefore, additional approaches are necessary if detailed knowledge on functional interactions is required.

### In‐depth functional characterization and spatial distribution

For an overall assessment of the composition of the microbial communities as well as the present functions, the above described methods can provide a comprehensive picture. Further, depending on the chosen technique, information at different levels of detail is obtained. For more spatial resolution, allowing an identification of the contributions of individual species and a restoration of potential pathways within the biofilm, within the planktonic community, or between both entities, a further in‐depth characterization is necessary.

Especially for biofilms, the spatial organization of the microbial cells is of highest relevance. As the general phylogenetic groups involved are already known after the analyses described above, more specific methods allowing individual cell labelling can resolve the biofilm organization (see Table 1). Most commonly, specific fluorescently labelled oligonucleotide probes for the rRNA targeting phylogenetic groups on the genera, family or phylum level can be developed or obtained from extensive public databases (e.g. http://probebase.csb.univie.ac.at). Standard protocols for the complete fluorescence *in situ* hybridization (FISH) procedure covering cell fixation, hybridization, washing and analysis are available (https://www.arb-silva.de/fish-probes). Nevertheless, greatest caution is needed when trying to utilize probes for uncultivated species in environmental samples, as here the optimization of the hybridization procedure has not been performed, yet (Amann and Fuchs, 2008) and protocol verification is therefore not possible. For the case study, the distribution of *Geobacteraceae* in the MFC could be of interest. The hypothesis above stated that the anode is pre‐enriched with *Geobacter* sp. In the following, additional species that contribute to organic acid conversion to acetate presumably settle on the biofilm. As starting point, a biofilm sample could be hybridized with a specific probe for *Geobacteraceae*, and all cells of the biofilm are stained with DAPI, a standard dye that binds to DNA. Using confocal laser scanning microscopy (CLSM), the visualization of the individually labelled microbial cells would indicate an enrichment of *Geobacteraceae* on the electrode surface, while the more outer layers of the biofilm would show the DAPI signal but not the hybridization signal of the *Geobacteraceae* probe. Further, this visual analysis would reveal the density of the cells within the biofilm. *Geobacter*‐dominated biofilms grown on acetate are often quite dense (Bond and Lovley, 2003; Virdis *et al*., 2012), while biofilms formed from complex wastewaters are characterized by a higher amount of extracellular polymeric substances and lower cell density. By adding further specific probes for the other members of the biofilm community, more detailed information on the spatial organization of the biofilm on a phylogenetic level and the number of the individual cells for each group can be revealed. While this information complements nicely the DNA‐based information detailed above, abundance differences for the same phylogenetic group using different methods (e.g., single cell based versus sequencing based) might appear. This can be assigned to the sample processing, as for instance the DNA‐based sequencing methods are partly biased due to different DNA extraction and amplification, and FISH can be biased due to fixation and hybridization efficiency differences (Amann and Fuchs, 2008; Krakat *et al*., 2017).

The next step for resolving the trophic networks and for understanding the individual functional contributions is a defined activity analysis complementing the information of potentially present functions based on DNA, RNA and protein analysis. As obvious from table 4 in Pant *et al*. (2013), the inflow composition in the MFC is not the same for all batches. Consequently, it would be interesting to resolve which species specifically contribute to the degradation in each batch and if the same species were able to perform the different functions. While a time resolved analysis can indicate the changes in abundance of different phylogenetic groups and could give a first indication about the different levels of activities, it does not reveal the actual activities. Spiking or replacing the substrate fraction with isotope labelled (e.g., ^13^C, ^15^N, ^34^S, ^2^H) substrate (here carboxylic acids), the fate of these substrates could be followed with advanced imaging technologies based on nanoSIMS (Musat *et al*., 2012; Chapleur *et al*., 2013). With this technique, the isotope signature of the substrate can be detected in the respective microbial cells and can be combined with analysis of the phylogenetic identity of these cells based on FISH. In combination with the continuous monitoring of current flow at the anode, the exact course of the substrate metabolization could be resolved in this low diversity biofilm. Afterwards, one could answer the question if it is only the acetate oxidation of *Geobacter* sp. that leads to current flow and which species convert other carboxylic acids to acetate. Alternatively, other species in the biofilm might also be electroactive and directly link the organic acid oxidation with current flow independent of a *Geobacteraceae* contribution (Koch *et al*., 2014b; Koch and Harnisch, 2016). Both cases can also evenly or unevenly coexist depending on the concentration of available substrates. These differences can only be revealed with highly resolved (single cell) in‐depth functional characterization of the biofilm (Table 1). Due to the costs of the labelled substrates, this kind of experiment is usually only performed on small scales (adequate for the current case study with a 25 ml of MFC). For bigger reactors, we recommend the set‐up of smaller microcosms based on the original reactor (see e.g., Pous *et al*. (2014)) for a more detailed functional analysis (Table 1). These can also include defined electrochemical measurements to further understand the underlying mechanisms of the microorganisms‐anode interaction (Pous *et al*., 2014).

## From reactor description to improved MET engineering

**Figure 3:**
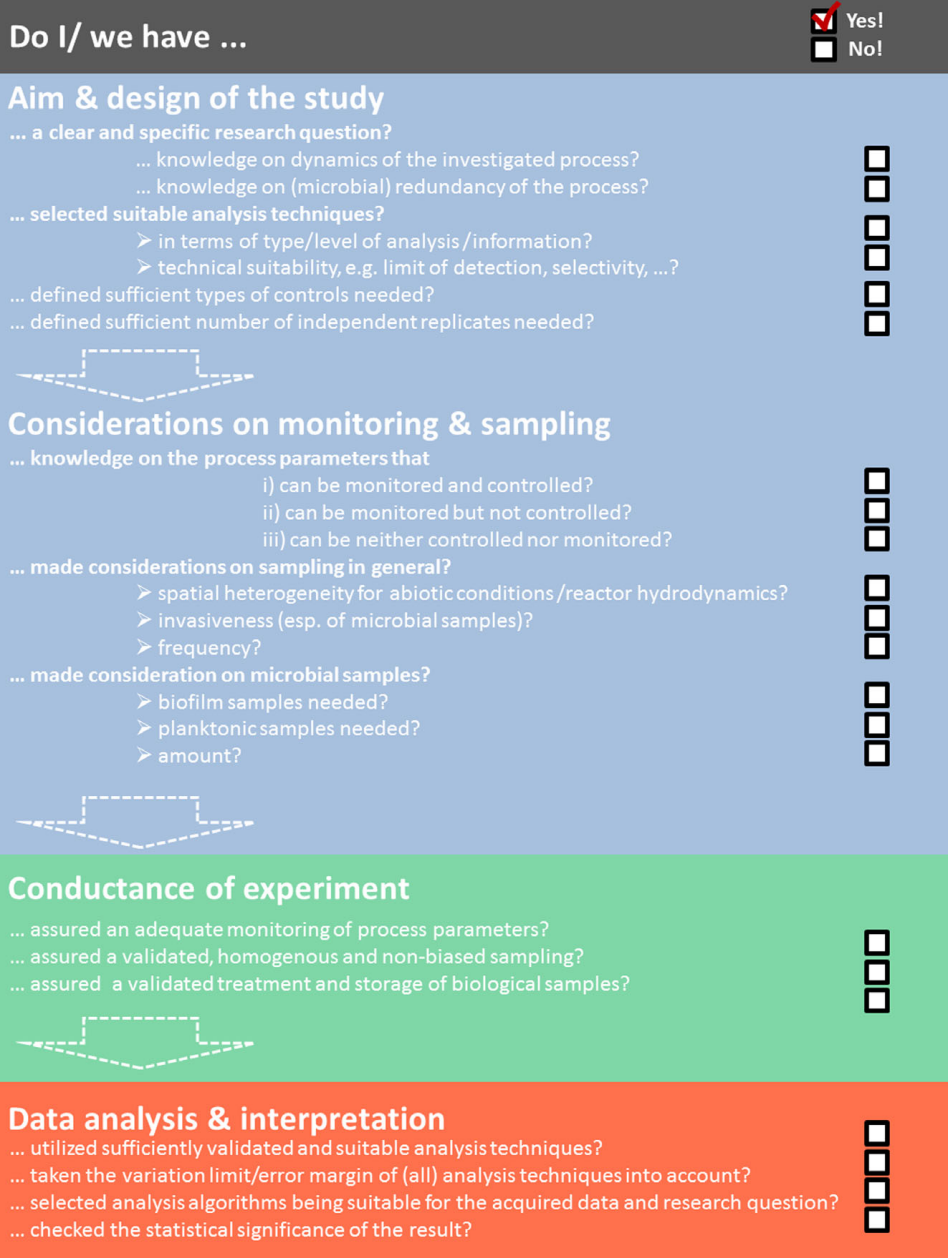
Checklist for planning, performing and data analysis of a microbiome‐based MET study: *Before experiment*: First, the framework of the study has to be set and identified, respectively, including the specific research question. Accordingly, the knowledge on the process has to be scrutinized. This concerns especially the dynamics of the microbial processes, the specificity of the investigated reactions and identification of potential relevant process parameters. The answers to these questions impact directly on the frequency of sampling and the analysing techniques to be used. Further, it is highly important to define the controls and number of independent replicates needed. In terms of controls, abiotic controls (with electrochemistry) as well as biotic controls (without electrochemistry) need to be considered at minimum. Depending on the research questions, further controls can be essential. For experiments as well as controls, it is highly important to have sufficient independent technical as well as biological replicates. In this context, sufficient means at least three replicates, but as microbiome‐based processes are depending on multiple variables, a higher number can be needed for sufficient statistical analysis. Here also considerations on the statistical method to be used later on are recommended (Cumming *et al*., 2007). *During experiment*: The guidelines for good scientific practice should be followed and especially proper sampling (without disturbing the process and microbiome (too much)), sample handling (e.g., oxygen tolerant sample, representative sample) and sample storage (e.g., immediately cooling after sampling, storage at −80°C especially for RNA and protein samples, stability of compounds to be determined) needs to be assured. The respective protocols (sampling as well as sample analysis) should be validated before the actual experiment starts and not varied during experiment. *After experiment*: First, the data acquired by a certain technique have to be checked on its validity as well as technical significance. If this can be assured and the techniques are still suitable for addressing the (maybe altered) research question, a suitable statistical analysis can be performed. Subsequently, the microbiome and its dependency on the process parameters are thoroughly analysed.

Looking at a single state of a bioreactor community gives only limited information regarding the diversity and corresponding functions (structure‐function relationship) for improvement strategies. This data only reveal that a specific inoculum, a specific substrate mixture and a specific reactor with a defined set of specific running conditions leads to a corresponding function. However, the reproducibility for similar set‐ups and conditions is not generally given. Microbiomes used as inocula are often not stable in their composition over time. Complex substrates usually vary over time, especially domestic wastewater shows even diurnal fluctuations in its composition (Martin and Vanrolleghem, 2014). Therefore, it takes more to understand the interrelation of a microbiome, its functions and their dependency on its specific BES environment. It is not sufficient to just observe a function. The aim is rather to proactively manage a microbiome based on variable process parameters to give their best functionality for a desired process or process sequence. A first step is the investigation of the community changes over time to get a better understanding on its dynamics (Fuhrman *et al*., 2015). Measuring representative samples in the same time frames as changes occur in the microbial community can help to identify the functional relevant microbial interactions but also relevant interactions between experimental conditions and the microorganisms. Aiming at a rapid data analysis, genomic approaches based on 16S rRNA gene sequencing are better suited for processing and comparing numerous samples than metagenomics approaches. Subsequently, multivariate analysis (e.g., using R package vegan (Oksanen *et al*., 2017) or the QIIME platform (Caporaso *et al*., 2010)) can be performed to explore the data and to identify underlying trends. The similarity between the samples can be visualized and a categorization of the samples regarding different primary parameters or changes after specific treatments becomes obvious. For a better interpretation, all primary and secondary parameters should be included (see also Fig. 3). This requires that all available experimental, and process data are determined for the time point of microbial sampling (obligatory!). Combining all measurements changes in the microbial community might go along with changes in certain primary and secondary parameters. Studying larger representative data sets with changes over a relevant range for each parameter, correlations between respective parameters and individual phylogenetic groups as well as their function can be identified (Ramette, 2007). They can further be combined with co‐occurrence analysis that shows which microorganisms are related in their appearance, for example always or never together, and association networks (e.g., CoNet App (Faust and Raes, 2016)). This information gives another indication for functional interactions in combination with the primary parameters that foster their occurrence and functionality. Also potential functional redundancies, that is, different microorganism (regarding their phylogenetic affiliation) are able to perform the same physiological function, can be revealed. Based on all these results, key players for certain process characteristics might be identified together with their optimal and limiting process conditions. This combined information is then used to develop specific microbiome steering and management strategies (Koch *et al*., 2014a) to boost desired functionalities and avoid system failures.

After this section, it is obvious that microbial ecology analyses cannot be performed with single samples or without the corresponding process data. It requires a reasonable experimental set‐up, representative sampling of the microorganisms as well as primary parameters covering all dynamics, and the suitable set of analysing techniques to get the most out of the data and to really understand the functional interconnections within the system. And as every technical system is individual, also the respective analysing strategies have to be carefully developed for each individual research question. A guide for developing such a strategy is provided in Fig. 3.

The practical realization of representative sampling is not trivial (Knight *et al*., 2012; Tickle *et al*., 2013), especially for biofilms. While planktonic cells are included in the reactor effluent and can be easily sampled, the biofilm is attached to the anode. Therefore, sampling is invasive. First, in most cases, the system has to be opened for sampling (i.e., to scrape of a small section of the biofilm or to remove a piece of the anode completely). While the first approach results in an electrode surface which can be newly colonized (and could therefore differ in its future biofilm composition compared to the existing biofilm and accordingly also provide a different functionality), the second approach reduces the available electrode surface area over time. Dependent on the reactor geometries and sampling intervals, this could impact the anode surface to reactor volume ratio and thus functionality of the biofilm attached to the electrode as well as the niche that the planktonic microbial community is facing. For both cases, one also has to be aware of potential differences in the electrode colonization based on mixing perturbations or certain flow regimes within the reactor resulting in spatial heterogeneity over the electrode (Pous *et al*., 2015). It is recommended to sample at different areas of the electrode and to compare the community composition (Dennis *et al*., 2013). If it is similar, later samples of only one electrode spot can be regarded as representative sample of the entire electrode biofilm community.

In the same way, the representability of a biological sample has to be confirmed, also the replicability of the whole system has to be ensured and proper control experiments need to be included. It is not sufficient to run a single reactor for significantly learning about the underlying principles for microbiome functions and their dependency on the primary parameters. Generating a sufficient data fundament in terms of replicates is possible for all laboratory scale experiments enabling a proper statistical analysis. In contrast, pilot scale experiments are more challenging and need careful assessment.

However, is not possible and also not recommended for every new experiment on microbial electrochemical technologies to apply advanced microbial ecology analyses. There are a number of general principles derived from microbial ecology that can be generally applied and can already substantially improve the system functions.

Starting from the general experimental set‐up: If the process of interest targets at the treatment of a complex waste stream (e.g., complex food waste, domestic wastewater), it is not appropriate to choose a defined mineral salt medium with a single or just a few defined carbon sources. This medium choice will always result in a microbial community with low complexity. This community, well adapted and specialized, will be highly functional in this individual experiment, but the results are not transferable to more complex or more diverse substrates to be treated in bioelectrochemical systems. If no process changes (e.g., hydraulic retention time, substrate composition, pH) are applied during the course of the experiment, the low diversity community will usually perform well. Especially, acetate‐based artificial wastewaters will be dominated by *Geobacter* sp. biofilms on the anode. They perform well in this ecological niche with best performance regarding coulombic efficiency (*CE*) and maximum current density (*j*_max_). However, they are highly inflexible when it comes to other substrates and could result in a complete system breakdown if the colonization with other microorganisms that convert the provided substrates to acetate is not fast enough. Therefore, pre‐enrichment of anodic biofilms with acetate‐based artificial wastewater is not recommended for most applications. If complex substrates are to be treated, it is superior to start the BES already from the beginning with complex representative substrate mixtures (Torres *et al*., 2007) or even the real substrate itself (e.g., domestic or industrial wastewater which is aimed to be treated, (Ishii *et al*., 2012; Rosa *et al*., 2017)). Combined with a highly diverse inoculum, it is very likely that a community capable of utilizing this mixture establishes during the starting period of operation. Although a highly diverse microbial community might in many cases not result in the best *CE* and *j*_max_ values (especially compared to *Geobacter* biofilms fed with acetate), their flexibility towards experimental changes and process variations as well as their stability regarding a certain function are the benefits of this strategy. A diverse microbiome is able to tolerate changes in the composition or loading rate of the provided substrates. Especially, if these happen in small steps, the microbiome will be able to adapt to changes over time and keep a stable functionality. An abrupt major change in the substrate composition is not recommended and would probably also affect a diverse microbial community leading to a temporary loss of functionality. But even in this case, it is very likely that the microbiome recovers (with an adapted community composition and function) and adapts to the new substrates over time and shows functional recovery. The trophic network within a complex microbiome is usually characterized by functional redundancy. That means that several substrate degradation pathways are present and utilized in parallel. Changes in the process regime can then lead to an intensification of certain pathways, while others are reduced. Variation of process conditions usually preserves different pathways. Even if they are currently silenced, they can be immediately upregulated if process conditions change. Shaping microbiomes for specific niches by lowering their diversity should be carefully considered as this often bears also several risks. For example, it can be advantageous to reduce the hydraulic retention time as well as pH for the exclusion of methanogenic archaea as it was performed in the case study and also proven in other studies (Sträuber *et al*., 2016; Hegner *et al*., 2017). But it has to be considered that reducing hydraulic retention times might also result in loss of slow growing microorganisms like syntrophic bacteria that are required for the degradation of certain organic acids. The knowledge about the presence, function and physiology of the microorganisms in a respective process is therefore necessary to choose the best‐suited process parameters when it comes to system improvement and optimization.

## Conclusions

Microbiome‐based processes are indispensable for future application of MET. Anodic processes represent a sustainable and energy‐saving alternative for the treatment of organic waste material. Furthermore, cathodic processes are also in development for future technical applications like sustainable production of chemical compounds and energy carriers. The challenge of microbiome‐based MET is founded in the connection of process engineering with the simultaneous maintenance of microbiome‐based functionality. Improving MET as black box systems based on a trial and error approach seems as little helpful as analysing microbiomes independent from the primary parameters. Comprehensive studies combining both aspects have to be the future guideline for MET improvement. As starting point for a reasonable experimental design, data acquisition and analysis, we suggest addressing a set of key questions (Fig. 3). These comprise a concerted experimental design, choice of microbial and analytical analysing techniques as well as data interpretation. In this way, the future challenges of MET development and optimization will be met based on proactive microbiome management.

## Conflict of interest

There is no conflict of interests.
